# Do We Need Multiple Informants When Assessing Autistic Traits? The Degree of Report Bias on Offspring, Self, and Spouse Ratings

**DOI:** 10.1007/s10803-015-2562-y

**Published:** 2015-09-03

**Authors:** Esmé Möricke, Jan K. Buitelaar, Nanda N. J. Rommelse

**Affiliations:** Department of Psychiatry, Nijmegen Centre for Evidence-Based Practice, Radboud University Nijmegen Medical Centre, P.O. Box 9101, 6500 HB Nijmegen, The Netherlands; Department of Cognitive Neuroscience, Donders Institute for Brain, Cognition and Behaviour, Radboud University Nijmegen Medical Centre, Nijmegen, The Netherlands; Karakter Child and Adolescent Psychiatry University Centre, Nijmegen, The Netherlands

**Keywords:** Autistic trait, Report bias, Self- and spouse-report, Parent-offspring effect, Preschooler and parent, General population

## Abstract

This study focused on the degree of report bias in assessing autistic traits. Both parents of 124 preschoolers completed the Social Communication Questionnaire and the Autism-spectrum Quotient. Acceptable agreement existed between mother and father reports of children’s mean scores of autistic traits, but interrater reliability for rank-order correlations was only fair. No evidence was found for report bias regarding parent-offspring autistic traits. However, adult autistic ratings were strongly biased: spouse-ratings were higher than self-ratings, correlations were only fair when both parents reported about the same person, and resemblance was higher for reports from the same person than for spouses’ separate self-reports. It is advisable to involve multiple informants when assessing autistic traits, and to use procedural and/or statistical remedies to control for report bias.

## Introduction

It has become quite common to collect information on psychopathology by asking informants and the person himself/herself to complete questionnaires. This has proven to be a time-efficient method and less expensive than administering structured interviews or conducting direct behavioural observations. The information gathered with questionnaires may be less comprehensive and thorough, but the user friendliness enables the collection of dimensional data from various persons. Use of multiple informants is recommended in developmental psychopathology, because each observant contributes unique information about internalizing and externalizing behaviour problems (De Los Reyes 2013; Dirks et al. 2012). This applies not only to children and adolescents (Achenbach et al. 1987; Duhig et al. 2000; Renk 2005; Stratis and Lecavalier 2015), but also to adults (Achenbach et al. 2005; Van der Ende et al. 2012). However, less is known about the utility and validity of questionnaire data and multiple informants in assessing autism spectrum disorders (ASD).

In most cases of ASD, information about a child’s autistic traits is reported by only one informant, generally the mother (for an overview of ASD screening questionnaires, see Fernandopulle 2011; García-Primo et al. 2014; Norris and Lecavalier 2010; Ozonoff et al. 2005; Zwaigenbaum et al. 2009). Information from the father and teacher is often missing, which can lead to a unilateral view of (problem) behaviour. Mothers, fathers, and teachers may interpret and evaluate certain behaviours differently due to unique personal experiences or situational specificity. Very little is known about the potential report bias that may affect ratings of autistic traits. Bias is a systematic error in measurement that may influence results and conclusions, and that can arise from selective recall, social desirability, interview situation and tools, question phrasing, answer alternatives, and/or digit preference (Fadnes et al. 2009; Podsakoff et al. 2003). Other informant characteristics, such as context, knowledge, experiences, personality, and/or psychopathology, may also determine the degree to which persons can give reliable information and mutual discrepancies exist (De Los Reyes and Kazdin 2005). For instance, persons who have ASD themselves may have another perception and interpretation of social behaviour, what justifies the question whether they are proper assessors of their own and others’ behaviour. These biases, in combination with task, ability, and motivational factors, may influence the informant’s behaviour in various stages of the response process, namely comprehension, retrieval, judgement, response selection, and response reporting (Podsakoff et al. 2003).

Bias can be based on two separate measures: agreement and reliability. Agreement refers to the extent to which raters assign exactly the same *absolute* scores to behaviour. Percentages give rough estimates of agreement, whereas Cohen’s kappa (*κ*) represents proportional agreement corrected by chance (Berry and Mielke 1988; Cohen 1960). Reliability points to the degree to which different raters estimate the same *relative* similarity of scores. Interrater reliabilities reflect proportional deviations from their means and can be expressed as correlation coefficients (Pearson product-moment, Spearman rank, intraclass), depending on the distribution of the data (i.e. the degree of normality) and the number of raters (i.e. two or several raters) (Multon 2010). Reliability parameters are most appropriate to distinguish persons in scientific research, but agreement parameters are preferable to measure behavioural changes in clinical practice (De Vet et al. 2006).

The most common method to examine report bias is to compare scores from different raters regarding the same subject (De Los Reyes and Kazdin 2005), for instance a mother and father reporting about the same child. Ideally, these ratings are congruent if they truly reflect the same construct. Alternatively, one can look at the relation between scores from the same rater regarding different subjects, for instance a mother rating her own child and a neighbour’s child. If these scores are correlated, report bias may likely play a role. There is a body of research on agreement of different informants on externalizing and internalizing problems (e.g. Achenbach et al. 1987, 2005; Duhig et al. 2000; Stratis and Lecavalier 2015; Van der Ende et al. 2012). For information on children’s functioning both parent and teacher reports are valuable, whereas for adults self-reports and spouse-reports are of more importance. However, is has not been established what the ‘better’ source of information is. It may be argued that the measure most strongly predicting outcome is the most valid one. A more safe conclusion is probably that a multi-informant approach is most optimal, because each observant contributes unique and specific information. This combination of observations may best reflect current functioning of the individual and his/her environment (Dirks et al. 2012; Renk 2005). In the context of ASD report bias has not elaborately been investigated in the general population.

Some studies have examined report bias of autistic traits by comparing ratings of multiple informants of the same subject. Posserud et al. (2006) concluded that there is little agreement between parents’ and teachers’ ratings of autistic traits using the Autism Spectrum Screening Questionnaire (ASSQ; Ehlers et al. 1999) in children from the general population. Similarly, Mattila et al. (2009) reported that agreement between informants was slight and that the correlation between parents’ and teachers’ scores on the ASSQ was weakly positive in population school children, and negative in high-scoring children. Constantino et al. (2007) used the Social Responsiveness Scale (SRS; Constantino and Gruber 2005) in a sample of children with and without pervasive developmental disorders. Correlations between scores of parents and teachers were significant for subscale and total scores: social awareness 0.66, social cognition 0.67, social communication 0.68, social motivation 0.57, autistic mannerisms 0.69, and total 0.72. When both informants reported elevated levels of autistic traits, the degree of diagnostic accuracy increased and the risk of report bias decreased. Bölte et al. (2008) determined maternal and paternal SRS scores in a normative and clinical sample of children. In the former, mean total scores differed moderately, yet significantly, but the correlation was strong (0.76). In the latter, mean total scores did not differ and were extremely high correlated (0.97). Kalyva (2010) assessed social skills in children with Asperger syndrome and normal controls, and found that the measures of agreement and reliability varied depending upon the composition of the group and the type of raters. Jepsen et al. (2012) investigated behavioural and emotional problems as well as social functioning in adolescents with ASD. Self, parent and teacher ratings were discrepant. The degree of agreement varied depending on the behaviours examined and the informants consulted.

All research groups emphasized the importance of assessment of autistic traits by different persons in various settings (at home, at school). This is especially relevant for children with ASD, because they experience more problems in the generalisation of skills across contexts and settings (Stratis and Lecavalier 2015). These studies regarding interrater agreement were performed among school children and adolescents in whom ASD pathology is more crystallised. As far as we know, similar research in preschool children has not been performed yet, whereas this is essential to get insight in autistic traits from an early age onwards. The Social Communication Questionnaire (SCQ; Berument et al. 1999) is considered as a valuable screener of autistic symptoms in young children (Eaves et al. 2006). It is built on the same items as the Autism Diagnostic Interview-Revised (ADI-R; Lord et al. 1994). However, information on interrater agreement on the SCQ is scarce.

Similarly, little is known about report bias influencing self-reported ratings on autistic traits of adults. The self-reported Autism-spectrum Quotient (AQ; Baron-Cohen et al. 2001) and the Broad Autism Phenotype Questionnaire (BAPQ; Hurley et al. 2007) are often used to obtain a proxy of adult autistic traits. However, studies comparing the self-report ratings with ratings of a significant other are limited. In three studies, self-report scores and spouse-report scores on the three subscales (social aloofness, pragmatic language, rigidity) and the total BAPQ were compared. Hurley et al. (2007) found that spouse-report scores were slightly, but not significantly, higher than self-report scores. However, they did not distinguish mothers from fathers, like Seidman et al. (2012) did. The latter study found that mothers’ self-reported aloofness was significantly higher and rigidity was significantly lower than husbands’ ratings about mother. Scores on the pragmatic language scale did not differ significantly. Fathers’ self-reported ratings versus wives’ ratings about father revealed no significant differences on any scale. Sasson et al. (2014) concluded that agreement between self-report and informant-report was moderate to strong when parents of children with ASD did not possess the broad autism phenotype (BAP) trait assessed, but that disagreement occurred when the parent scored positive on the trait rated. Especially fathers showed selective blind spots in self-reports which may lead to underestimation of BAP traits. Studies in a related area (attention-deficit/hyperactivity disorder, ADHD) also show that agreement between self and significant-other reports may often be questionable (Alexander and Liljequist 2013; Katz et al. 2009; Kooij et al. 2008). Thus, when studying adult ratings, additional information from a significant other is desirable and vital. Moreover, given that ASD is a highly heritable disorder (Sucksmith et al. 2011) and subthreshold symptoms may often exist in parents of children with ASD (Maxwell et al. 2013; Sasson et al. 2013; Wheelwright et al. 2010), there may be a relation between self-report and report about offspring due to report bias and not only because of familiality of autistic traits. Thus far, no studies have examined this issue.

Clarifying to which degree report bias influences reports on autistic traits is important for two reasons. In scientific research, one should consider that heritability of autistic traits based on questionnaires may be over- or underestimated. In clinical practice, one should realize that sole reliance on maternal reports (in children) or self-reports (in adults) may give an inaccurate or incomplete picture of autistic behaviour. Therefore, we aimed to investigate systematically the degree of report bias in parental reports concerning autistic traits, not only in their child, but also in themselves and their spouse. Further, we aimed to examine differences in the correlations between parent and offspring autistic traits according to father and mother. Both parents from 124 families selected from a population based sample were asked to complete a measure of autistic traits regarding their child (SCQ), themselves (AQ self-report), and their spouse (AQ spouse-report). The subsample selection was based on an almost equal division of children with low, moderate, and high autistic scores. We assumed that both agreement and reliability between raters may be higher if autistic traits are clinical compared to subthreshold or non-clinical, because maternal and paternal ratings tended to correspond more when it concerns clearly observable problematic behaviour (Duhig et al. 2000).

## Methods

### Participants

The Medical Ethics Committee of University Medical Centre Utrecht approved the study. We contacted a subsample (*N* = 188) from a general population birth cohort of children born between August 2000 and August 2001 in the province of Utrecht, The Netherlands. Children were selected on the basis of scores on the Early Screening of Autistic Traits Questionnaire (ESAT; Dietz et al. 2006; Swinkels et al. 2006), which was administered at the age of 14–15 months. This questionnaire consists of 14 items with “yes” (1) and “no” (0) answers. A low score represents normal behaviour, higher scores indicate more autistic traits. Generally, children with a score of three or more are considered to be screen-positive and thus at high risk for developing ASD. This cut-off detected 0 % of a non-selected sample, and 90.1 % of the children with ASD (Swinkels et al. 2006). However, we used a cut-off of two or more, because we were also interested in children with milder autistic traits.

**Fig. 1:**
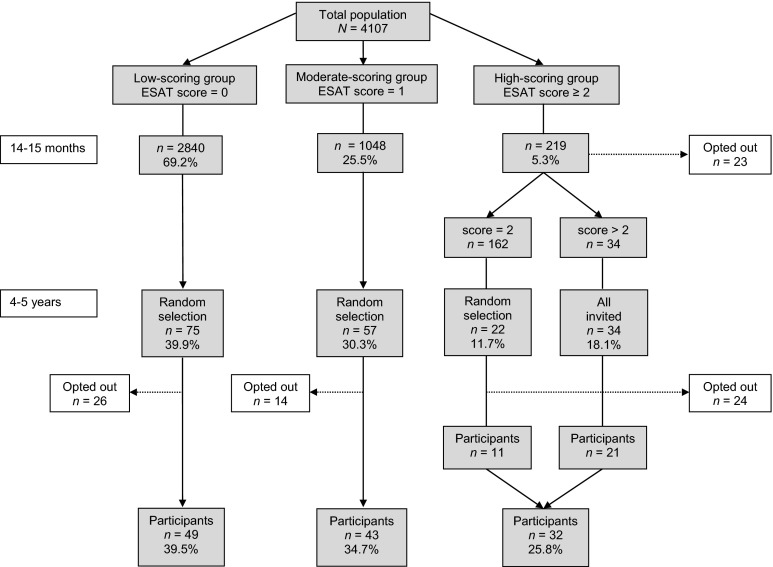
Flow chart of participants

Children with low (0) and moderate (1) ESAT scores were randomly selected. Children with high (≥2) ESAT scores were approached in phases. First, we invited all children with scores of 3 or more, followed by a random selection of children with scores of 2. Not all the selected families could be reached or were willing to participate in the follow-up at age 4–5 years. Finally, 124 out of 188 families (66.0 %) consented to participate after a complete explanation of the procedure. The final division over the ESAT scoring groups was as follows: low 39.5 %, moderate 34.7 %, and high 25.8 %. The last group included 21 children with a score ≥3. See the flow chart in Fig. 1.

The SCQ was filled in by 119 fathers and 124 mothers. Concerning the AQ, fathers completed 120 self-reports and 116 spouse-reports; mothers completed 124 self-reports and 119 spouse-reports. The mean age of the participants was: fathers 39.2 years (*SD* = 4.2; range 27.7–48.1), mothers 36.8 years (*SD* = 3.9; range 25.3–48.2), boys 52.9 months (*SD* = 4.6; range 42.9–67.6), girls 52.4 months (*SD* = 4.9; range 42.3–66.1). In this sample, boys (*n* = 73; 58.9 %) were overrepresented in comparison to girls (*n* = 51; 41.1 %), but the proportion of boys and girls was similar in each scoring group. Five children (3.9 %) were known to be developmentally delayed; three boys had a formal ASD diagnosis, and two girls had multiple handicaps.

Because access to information about non-responders was not allowed, we investigated possible selection bias by comparing the data of responders with demographic data for the general population (Central Office for Statistics 2003). Of the children in the sample, 93.5 % was Dutch (*n* = 116) and 4.0 % non-Dutch (*n* = 5). The nationality of 2.4 % of the sample was not known (*n* = 3). Our sample contained more Dutch children than the population average (82.1 %). A relatively large number of parents had a high educational level (college or university degree) compared to persons in the population (mothers 41.1 vs. 38.9 %; fathers 50.0 vs. 36.0 %). The socioeconomic status (SES), based on mean level of education and occupation of both parents, varied from low (*n* = 15; 12.1 %) through moderate (*n* = 49; 39.5 %) to high (*n* = 57; 46.0 %); in 2.4 % (*n* = 3) of the cases SES was unknown. Families with low SES were underrepresented and families with high SES were overrepresented.

### Instruments

Both parents were asked to fill in three questionnaires regarding autistic traits: about their child (SCQ), themselves (AQ self-report), and their spouse (AQ spouse-report).

### Social Communication Questionnaire

Autistic traits at age 4–5 years were measured with the SCQ Current Version (Berument et al. 1999), in which parents reported about their child’s behaviour in the last three months. It consists of 40 items covering four domains: reciprocal-social interaction, language and communication, repetitive and stereotyped behaviour, and other behaviour. Twenty-five items were reversely coded, so that typical behaviour is scored as 0, and the lack of competences or the experience of problems is rated as 1. Maximally two missing values per domain and four in total were allowed (<10 % of 40 items). These missing values were replaced by the individual domain means of the parent in question (i.e. his/her domain score divided by the number of completed items). The minimum total score is 0 and the maximum total score is 34 or 39, depending on the absence or presence of language and speech respectively. The official cut-off for ASD is fixed at 15, but for younger children a lower cut-off of 11 seems to be more accurate (Allen 2007; Corsello et al. 2007). However, for most analyses we focused on the continuous scores.

### Autism-spectrum Quotient

Autistic traits of the parents were assessed with the Dutch self-report and spouse-report versions of the original AQ (Baron-Cohen et al. 2001), consisting of 50 statements regarding social skills, attention switching, attention to detail, communication, and imagination. In the English version, the two agree- and two disagree-answers are generally dichotomized. Answers pointing at autistic traits receive a score of 1, resulting in a total score between 0 and 50. The original cut-off for ASD in males and females was set at 32, but other cut-offs are also utilized (for review see Ruzich et al. 2015). In non-clinical samples a mean cut-off of 18 for males and 15 for females was computed. This dichotomous scoring method and these accompanying cut-offs were used to compute agreement between groups with low and high scores on autistic traits. However, in the Dutch version, items are commonly scored on a four-point Likert scale: 1 definitely agree, 2 slightly agree, 3 slightly disagree, 4 definitely disagree. Twenty-four items in which agree-answers were characteristic for autism were reversely coded. If the maximum number of missing values was less than two per scale and five in total (<10 % of 50 items), these missing values were replaced by the individual scale means of the reporting parent (i.e. his/her scale score divided by the number of completed items). The minimum total score is 50 and the maximum total score is 200. For most analyses, we considered this full range of scores in line with previous studies (Austin 2005; Hoekstra et al. 2008), which best resembles a broad continuum from normal to deviant behaviour.

### Statistical Analyses

SCQ data showed a skewed distribution of scores. The distributions of dichotomous total scores (i.e. below and above the cut-off) of father and mother were compared with Chi square test (*χ*^*2*^) and the measure of interrater agreement was determined (*κ*). Wilcoxon Signed Ranks Tests were used to examine mean differences in continuous SCQ ratings. For further analyses, SCQ ratings were normalized using a Van der Waerden transformation. Intraclass correlation coefficients (*ICCs*) were calculated to examine interrater reliability corrected for similarity based on chance. Thereafter, partial correlations (*pr*), corrected for the mean total self-report AQ score of both parents, were computed to examine whether parental autistic traits influenced offspring ratings. Differences between correlations with (*pr*) and without (*ICCs*) correction for parental ratings were calculated using Fisher’s r-z transformed analyses.

AQ data showed a normal distribution of scores. Chi square tests (*χ*^*2*^) were computed for the distributions of dichotomous total scores regarding mother (mother about self and father about mother) and regarding father (father about self and mother about father), and measures of interrater agreement (*κ*) were established. Means and standard deviations of continuous scores of self- and spouse-reports were computed. We distinguished three types of combinations of AQ reports: (1) self-reports (mother about self with father about self), (2) reports *about* the same person (mother about self with father about mother, and father about self with mother about father), and (3) reports *from* the same person (mother about self with mother about father, and father about self with father about mother). Paired samples *t* tests were executed to explore the measure of absolute agreement between scores. General Linear Models (GLMs) repeated measures analyses were used to examine main and interaction effects of gender and reporter. *ICCs* were calculated to determine the relative position of parental scores, and Williams’s T2 statistic was used to compare various *ICCs*.

Relations between child and parent autistic ratings were calculated using Spearman’s rank correlation coefficients for the corresponding constructs of SCQ and AQ. Fisher’s r–z transformed analyses were used to compute differences in parent-offspring correlations according to the parent him/herself and his/her spouse. The minimum level of significance was defined as *p* ≤ 0.05. *ICCs* were interpreted according to the guidelines as described by Cicchetti (1994): poor (<0.40), fair (0.40–0.59), good (0.60–0.74), and excellent (0.75–1.00). Cohen’s *κ* measures of agreement were regarded as poor (<0.00), slight (0.00–0.20), fair (0.21–0.40), moderate (0.41–0.60), substantial (0.61–0.80), and almost perfect (0.81–1.00) (Landis and Koch 1977). Cohen’s *d* effect sizes were considered as small (0.20), medium (0.50), and large (0.80) (Cohen 1992). The statistical software package IBM SPSS Statistics 20 (IBM Corp. 2011) was used.

## Results

Only SCQ and AQ total scores are described here. Please see tables and figures for results regarding domains and scales.

### Degrees of Report Bias on Estimates of Autistic Traits in Offspring

Parental reports about dichotomous SCQ scores below and above the cut-off (11) were in concordance with each other (*χ*^*2*^ < 0.001). In 79.0 % both parents assigned a low score, and in 5.9 % a high score. In 15.1 % the parents disagreed, with 9.2 % of the fathers scoring above the cut-off whereas mothers were scoring below, and with 5.9 % of the mothers rating above the cut-off whereas fathers were rating below. However, the measure of interrater agreement after correction for chance was only fair (*κ* = 0.35). The distributions of continuous SCQ total scores were right-skewed (skewness: *ƴ*_*1*_ mother = 2.29; *ƴ*_*1*_ father = 2.36) and quite peaked (kurtosis: *ƴ*_*2*_ mother = 8.07; *ƴ*_*2*_ father = 9.33). Therefore, non-parametric tests were used. Mean SCQ total scores of both parents did not differ significantly (mother *M* = 6.39, *SD* = 4.76; father *M* = 6.90, *SD* = 5.34; *z* = −1.38, *p* = 0.17). However, the *ICC* between maternal and paternal scores was only fair (*ICC* = 0.58; *p* < 0.001). The partial correlation was not influenced by the own AQ scores of both parents (*pr* = 0.57, *p* < 0.001; *z* = 0.05, *p* = 0.96). See Table 1.

**Table 1:** Means, standard deviations, differences, and correlations of SCQ scores

SCQ	Mother *N* = 124	Father *N* = 119	Difference between means of father and mother (*z* ^a^) and *p* value (*p*)	Intraclass correlation coefficient between parental reports *ICC* ^d^
Domain	*M* (*SD*)	*M* (*SD*)		
Interaction	1.39 (1.91)	1.50 (2.09)	−0.51^b^ (.61)	0.55**
Communication	3.03 (1.91)	2.99 (1.83)	−0.19^c^ (.85)	0.51**
Behaviour	1.77 (1.86)	2.13 (2.01)	−1.87^b^ (.06)	0.42**
Others^e^	0.19 (0.45)	0.23 (0.51)	−1.00^b^ (.32)	0.50**
Total	6.39 (4.76)	6.90 (5.34)	−1.38^b^ (.17)	0.58**

** *p* < 0.01

^a^Wilcoxon Signed Ranks Test

^b^Based on negative ranks

^c^Based on positive ranks

^d^Intraclass correlation coefficient computed after Van der Waerden’s transformation

^e^The domain *Others* includes three items regarding current language level, self injury, and attention to voice

### Degrees of Report Bias on Estimates of Autistic Traits in Parents

**Fig. 2:**
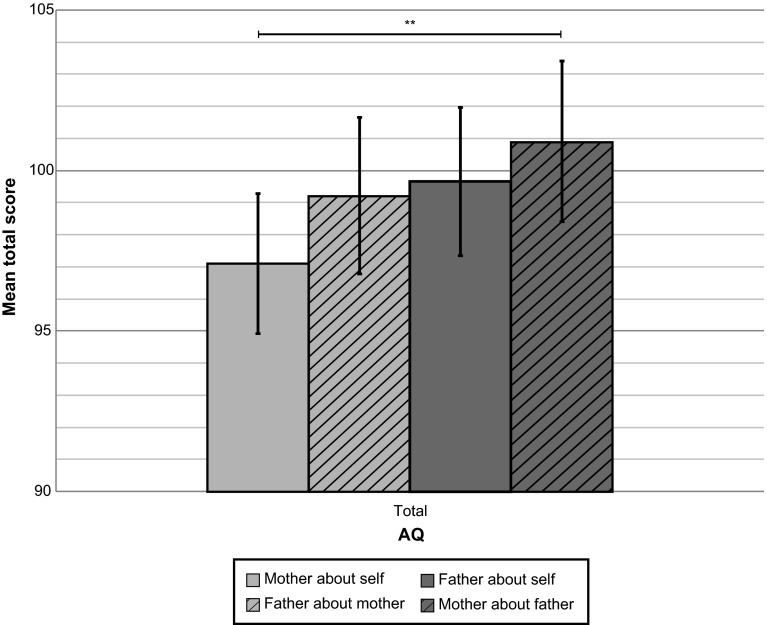
Bar chart with means, 95 % confidence intervals, and significant differences of AQ total scores of self- and spouse-reports illustrating report bias. *Note* significant result of paired samples *t* test: ** *p* ≤ 0.01

**Fig. 3:**
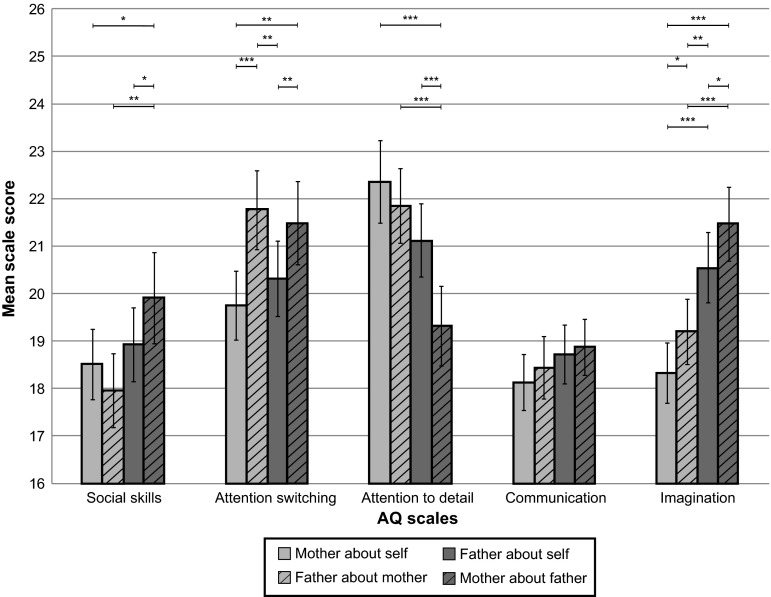
Bar chart with means, 95 % confidence intervals, and significant differences of AQ scale scores of self- and spouse-reports illustrating report bias. *Note* significant results of paired samples *t* tests: * *p* ≤ 0.05; ** *p* ≤ 0.01; *** *p* ≤ 0.001

The distributions of dichotomous AQ scores showed modest agreement. In only 65.5 % parents agreed on the scores regarding mother (with 43.1 % both scoring below and 22.4 % both scoring above the cut-off), but in 34.5 % parents disagreed on maternal autistic traits (with 18.1 % spouse-ratings above but self-ratings below the cut-off; and in 16.4 % self-ratings above and spouse-ratings below the cut-off) (*χ*^*2*^ = 0.003). In 70.7 % parents agreed on the scores regarding father (with 56.9 % both scoring below and 13.8 % both scoring above the cut-off), but in 29.3 % parents disagreed on paternal autistic traits (with 20.7 % spouse-ratings above but self-ratings below the cut-off; and in 8.6 % self-ratings above and spouse-ratings below the cut-off) (*χ*^*2*^ = 0.001). The measures of interrater agreement after correction for chance were only fair (scores regarding mother *κ* = 0.28; scores regarding father *κ* = 0.29). The continuous AQ total scores were distributed normally; there were no indications for extreme measures of skewness *ƴ*_*1*_ and kurtosis *ƴ*_*2*_ (mother about self: *ƴ*_*1*_ = 0.09, *ƴ*_*2*_ = −0.66; father about self: *ƴ*_*1*_ = 0.47, *ƴ*_*2*_ = 1.02; mother about father: *ƴ*_*1*_ = 0.61, *ƴ*_*2*_ = 0.24; father about mother: *ƴ*_*1*_ = 0.08, *ƴ*_*2*_ = −0.53). The means and standard deviations of AQ total scores were as follows: mother about self *M* = 97.10, *SD* = 12.41; father about self *M* = 99.65, *SD* = 12.89; mother about father *M* = 100.90, *SD* = 13.91, father about mother *M* = 99.22, *SD* = 13.40. GLM indicated that there was no significant effect of gender on AQ total scores (*F* = 2.65, *p* = 0.11, *d* = 0.31). However, there was a main effect of reporter: spouse-reports revealed higher AQ scores than self-reports (*F* = 7.41, *p* < 0.01, *d* = 0.51). No interaction between gender and reporter was present (*F* = 0.36, *p* = 0.55, *d* = 0.11), suggesting both parents tended to attribute higher AQ scores to their spouse than to themselves (Tables 2, 3; Figs. 2, 3).

**Table 2:** Means and standard deviations of AQ scores

Scales	Mother about self *N* = 124 *M* (*SD*)	Father about self *N* = 120 *M* (*SD*)	Mother about father *N* = 119 *M* (*SD*)	Father about mother *N* = 116 *M* (*SD*)
Social skills	18.51 (4.24)	18.93 (4.36)	19.91 (5.35)	17.96 (4.31)
Attention switching	19.76 (4.13)	20.32 (4.40)	21.49 (4.91)	21.77 (4.59)
Attention to detail	22.36 (4.91)	21.13 (4.30)	19.32 (4.68)	21.86 (4.33)
Communication	18.14 (3.36)	18.72 (3.43)	18.87 (3.33)	18.44 (3.60)
Imagination	18.33 (3.59)	20.55 (4.13)	21.47 (4.36)	19.20 (3.82)
Total	97.10 (12.41)	99.65 (12.89)	100.90 (13.91)	99.22 (13.40)

**Table 3:** General linear model repeated measures of AQ scores

AQ		Main effect of reporter self-spouse		Main effect of gender father-mother		Interaction effect of reporter and gender	
Scales	*df*	*F*	*d*	*F*	*d*	*F*	*d*
Social skills	1,112	0.21	0.09	6.54**	0.48	4.96*^a^	0.42
Attention switching	1,113	27.46**	0.98	0.05	0.04	1.91	0.26
Attention to detail	1,113	11.05**	0.62	9.80**	0.59	5.33*^b^	0.43
Communication	1,112	0.29	0.10	2.45	0.29	0.10	0.06
Imagination	1,111	14.77**	0.73	27.98**	1.00	0.01	0.02
**Total**	1,111	7.41**	0.51	2.65	0.31	0.36	0.11

Significant interaction effects of reporter and gender were found for two AQ scales: social skills (*p* = 0.03) and attention to detail (*p* = 0.02). Post hoc tests revealed that ^a^ for social skills father ascribed *higher* scores to himself than to his spouse (*t* = 1.83, *p* = 0.07) and mother ascribed *lower* scores to herself than to her spouse (*t* = −2.60, *p* = 0.01), whereas ^b^ for attention to detail father gave *lower* scores to himself than to his spouse (*t* = −1.12, *p* = 0.27) and mother gave *higher* scores to herself than to her spouse (*t* = 4.67, *p* < 0.001). Thus according to both spouses, father seems to have less social skills than mother, whereas mother seems to show more exceptional attention to details

*d* Cohen’s *d* effect size: 0.2 = small; 0.5 = medium; 0.8 = large

* *p* ≤ 0.05; ** *p* ≤ 0.01

*ICCs* between self-report and spouse-report about the same person were only fair (about mother *ICC* = 0.53, *p* ≤ 0.001; about father *ICC* = 0.59; *p* ≤ 0.001). The *ICC* between spouses using self-reports was poor (*ICC* = 0.26, *p* < 0.01), indicating that spouses hardly resembled each other in autistic traits. In contrast, *ICCs* between spouses when using scores from the same person were somewhat higher (from mother *ICC* = 0.36, *p* < 0.001; from father *ICC* = 0.42; *p* < 0.001), albeit not or hardly significant (self-reports vs. maternal reports *t* = 1.32, *p* = 0.19; self-reports vs. paternal reports *t* = 1.94, *p* = 0.05). See Table 4.

**Table 4:** Correlations and differences therein of various combinations of AQ scores

AQ	Self-reports	Reports *from* the same person				Reports *about* the same person	
	Mother about self-Father about self	Mother about self-Mother about father	Difference between correlations; self-reports vs. maternal reports	Father about self-Father about mother	Difference between correlations; self-reports vs. paternal reports	Mother about self-Father about mother	Father about self-Mother about father
Scales	*ICC* ^a^	*ICC* ^a^	*t* ^b^	*ICC* ^a^	*t* ^b^	*ICC* ^a^	*ICC* ^a^
Social skills	0.33**	0.25*	−1.07	0.30**	−0.30	0.50**	0.63**
Attention switching	0.15*	0.10	−0.55	0.13	−0.28	0.47**	0.42**
Attention to detail	0.00	−0.05	−0.45	−0.03	−0.35	0.47**	0.39**
Communication	0.03	0.19*	1.67	0.36**	3.25**	0.30**	0.47**
Imagination	0.14*	0.14*	0.06	0.23**	1.09	0.53**	0.52**
Total	0.26**	0.36**	1.32	0.42**	1.94	0.53**	0.59**

* *p* ≤ 0.05; ** *p* ≤ 0.01

^a^Intraclass correlation coefficient

^b^Williams’s T2 statistic

### Effects of Report Bias on Estimates of Parent-Offspring Relations Regarding Autistic Traits

When mother rated the autistic traits of herself and her child, a small but significant correlation was observed (*r* = 0.23, *p* < 0.01), which was similar when father rated the autistic traits of mother and child (*r* = 0.20; *p* = 0.03). In contrast, no significant correlations between the total number of fathers’ and offspring autistic traits were found when using father ratings (*r* = 0.16; *p* = 0.08) or mother ratings (*r* = 0.15; *p* = 0.10). In both cases, the difference between self and spouse ratings was not significant (about mother and child: *z* = 0.30, *p* = 0.76; about father and child: *z* = 0.11, *p* = 0.91), indicating no report bias.

## Discussion

Both parents of 124 preschool children, selected from a general population sample, completed validated and wide-spread questionnaires regarding autistic traits. We examined the degree of report bias in parental reports concerning these traits in their child, in themselves and in their spouse, and in the parent-offspring correlation. The results indicated that there was acceptable agreement, but only fair interrater reliability between paternal and maternal reports on autistic traits in the child (SCQ). No evidence for report bias was found for the relation between parent-offspring autistic traits. In contrast, adult autistic traits (AQ) were strongly influenced by report bias. Thus, parent’s own autistic traits merely seemed to affect their self- and spouse-reports, and not the reports concerning autistic traits in their child.

Although no evidence was found for report bias on the mean total scores of autistic traits of children, the scores showed only fair interrater reliability. In 15.1 % the parents disagreed, with 9.2 % of the fathers scoring above and mothers scoring below the cut-off, and with 5.9 % of the mothers rating above and fathers rating below the cut-off. This is in contrast with the high scores of interrater reliability on the SRS between fathers and mothers as found by Constantino and Gruber (2005) (*ICC* = 0.91) and by Bölte et al. (2008) (*ICC* = 0.76 normative sample; *ICC* = 0.97 clinical sample). However, this seems to correspond with previous studies, showing no significant differences in mean scores, yet poor reliability between paternal and maternal ratings of ASD (Kalyva 2010) and other child psychiatric disorders (internalizing, externalizing) (Moreno et al. 2008). If mean differences occur, it is often mother who reports more symptoms in her child than father (Caye et al. 2013; Langberg et al. 2010; Mascendaro et al. 2012; Sollie et al. 2013). Our findings indicate the absence of systematic overreporting or underreporting of autistic traits by mother or father, since the mean scores at group level are about similar. However, the relative ranking of children in terms of number of autistic traits would be rather different when performed by mothers than by fathers. This may be explained by the hypothesis that the rating of autistic traits may be somewhat more complicated than that of externalizing or internalizing symptoms. This would fit with the results of Stratis and Lecavalier (2015) who found that parent–parent agreement was higher for externalizing (*r* = 0.71) and internalizing problems (*r* = 0.69) than for social skills (*r* = 0.47). First, many autistic traits are formulated at a more abstract level than the better observable externalizing and internalizing symptoms. Second, rating aspects of interaction and communication interferes with and co-depends on the mutual social behaviour between child and parents. These two factors make the rating of autistic traits more sensitive to interpretation and thus bias. As a consequence, in clinical practice, where the mean score is most important, a SCQ filled in by one parent seems to be sufficient. However, obtaining a second parental report may be advised as an extra source of information, particularly in less clear cases. For research purposes it may be recommended to acquire scores on a child’s autistic traits from fathers, mothers, teachers and/or closely involved others when making a relative ranking.

No evidence was found for report bias with regard to familiality of autistic traits: both parents reported somewhat more resemblance between mother and child than between father and child, albeit all correlations were low. This is different from earlier results of absent significant correlations between the child’s social communication score and the parents’ BAPQ score (Seidman et al. 2012), and opposite to findings that social responsiveness scores of children correlated stronger with BAPQ scores of fathers than of mothers (Maxwell et al. 2013). Even though our study and previous studies (Baron-Cohen et al. 2001; Bishop et al. 2004; Wheelwright et al. 2010) indicate that female autistic scores are lower than male autistic scores, our result that children resemble their mother more than their father in terms of autistic traits, may imply that these traits have a stronger female aetiology than previously assumed. It is speculated that women can have autistic traits, expressed in a different phenotype than in men, which is not always properly ascertained (Van Wijngaarden-Cremers et al. 2014). This finding may be informative for phenotypic and genetic studies in ASD (Maxwell et al. 2013; Sasson et al. 2013) as well as in clinical practice, where the assessment of mother’s autistic traits may be of particular importance. Autistic traits of both mother and father should be considered to get a more nuanced and reliable picture of autistic traits transmission.

Clear evidence for report bias was present when examining adult autistic ratings: (1) spouse ratings were significantly higher than self ratings, (2) modest agreement and only fair interrater reliability was observed between self- and spouse-reports *about* the same person (in 34.5 % parents disagreed on maternal autistic traits and in 29.3 % parents disagreed on paternal autistic traits), and (3) autistic resemblance between spouses was somewhat higher (i.e. ‘overestimated’) for the comparison of reports *from* the same person than for the comparison of spouses’ separate self-reports. These results differ from those of Seidman et al. (2012), who did not find significant differences on self- and spouse-report total BAPQ scores, but partly correspond with those of Hurley et al. (2007), showing that spouse-reports were slightly higher than self-reports, and that self- and spouse-report correlations of total BAPQ scores were poor for autistic parents (*r* = 0.39) and moderate for control parents (*r* = 0.66). Sasson et al. (2014) distinguished between parents with and without the BAP traits being assessed. Significant and positive agreement existed between self- and spouse-reports of parents without BAP traits. However, selective disagreement occurred when parents positive on specific BAP traits filled in self-reports, but this did not extend to spouse-reports. Our findings suggest that either self-reported autistic ratings *underestimate* or spouse-ratings *overestimate* the true degree of autistic traits present, leading to false negatives and false positives respectively. Further studies are needed to increase knowledge about this issue and the consequences for scientific research and clinical practice.

This study had some limitations. First, the sample was relatively small, and there were more boys than girls, as well as children from higher SES families. Second, parents were instructed to fill in the questionnaires independently. Nevertheless, parents may have discussed about certain items. Consequently, the real degree of report bias may be even higher than estimated in our study. Third, unfortunately, no other persons were consulted. Although parents and spouses can give valuable information for screening purposes and may be the first to recognize problems, their reports may not be as accurate and effective as clinical observations to identify behavioural abnormalities, especially not when they show many autistic traits or suffer from other psychological problems themselves. Reporting bias can be decreased by involving professionals who judge behaviour more objectively based on standardised measurements and clinical experience. Thus reports of different informants should be considered as complementary. Fourth, the SCQ is seen as the ‘gold’ screening instrument for autistic symptoms in both epidemiological and clinical research, even in very young children. However, there are some new population measures for autistic traits, like the AQ-Child (Auyeung et al. 2008) and the SRS-2 (Constantino and Gruber 2012), which also may be useful. Fifth, the mutual agreement between SCQ and AQ scores of children and parents respectively may be limited due to differences in content, phrasing, and scoring of items. Using both the child and adult version of either the AQ or the SRS-2 is advisable in future research.

In summary, this study indicates that the rating of autistic traits is just as susceptible to report bias as ratings of other psychiatric problems. If confirmed in larger studies, it is advisable to use procedural and/or statistical remedies to control for report bias in scientific research (De Los Reyes 2013). In addition, it is desirable to determine how many informants would be necessary or sufficient, and to establish the relevance of their contributions, so that the collection of data is optimal (Smith 2007). This will also enable to answer the rhetorical question posed in our title. Now, we could not specify the exact number and type of informants. The design of this study was not suitable for this purpose. For children we only had reports from father and mother; for parents we only had self- and spouse-reports. Data observed and reported by significant others (e.g. (grand)parents, siblings, teachers, clinicians) were missing. Unfortunately, weighting the relevance of the parental contributions was not possible either, because we did not have so called ‘gold standards’ with which the results could be compared. Nevertheless, our answer is: yes, several informants are needed in reporting about autistic traits in children and adults. More research into valid autistic measures to be filled in by other informants is essential, so that these additional measures can be used more often in diagnosing ASD. Teachers see a child regularly in both structured and natural social situations, and may have a better sense of what is typical behaviour than parents. Parents, siblings, or spouses of adults may also be able to report behaviour more objectively than the adult him/herself. The involvement of multiple informants in screening- and diagnostic procedures in clinical practice is preferable (Dirks et al. 2012; Renk 2005). The fact that reports from different informants about the same individual do not completely correspond with each other may cause confusion. However, these reports should not be considered as contradictory, but as complementary, because each informant may interpret and evaluate certain behaviours differently due to unique personal experiences or situational specificity.

## References

[CR1] Achenbach TM, Krukowski RA, Dumenci L, Ivanova MY (2005). Assessment of adult psychopathology: Meta-analyses and implications of cross-informant correlations. Psychological Bulletin.

[CR2] Achenbach TM, McConaughy SH, Howell CT (1987). Child/adolescent behavioural and emotional problems: Implications of cross-informant correlations for situational specificity. Psychological Bulletin.

[CR3] Alexander, L., & Liljequist, L. (2013). Determining the accuracy of self-report versus informant-report using the Conners’ Adult ADHD Rating Scale. *Journal of Attention Disorders. *doi:10.1177/1087054713478652. 10.1177/1087054713478652 23503811

[CR100] Allen CW, Silove N, Williams K, Hutchins P (2007). Validity of the Social Communication Questionnaire in assessing risk of autism in preschool children with developmental problems. Journal of Autism and Developmental Disorders.

[CR4] Austin EJ (2005). Personality correlates of the broader autism phenotype as assessed by the Autism Spectrum Quotient (AQ). Personality and Individual Differences.

[CR5] Auyeung B, Baron-Cohen S, Wheelwright S, Allison C (2008). The Autism Spectrum Quotient: Children’s Version (AQ-Child). Journal of Autism and Developmental Disorders.

[CR6] Baron-Cohen S, Wheelwright S, Skinner R, Martin J, Clubley E (2001). The Autism-Spectrum Quotient (AQ): Evidence from Asperger syndrome/high-functioning autism, males and females, scientists and mathematicians. Journal of Autism and Developmental Disorders.

[CR7] Berry KJ, Mielke PW (1988). A generalization of Cohen’s kappa agreement measure to interval measurement and multiple raters. Educational and Psychological Measurement.

[CR8] Berument SK, Rutter M, Lord C, Pickles A, Bailey A (1999). Autism Screening Questionnaire: Diagnostic validity. British Journal of Psychiatry.

[CR9] Bishop DVM, Maybery M, Maley A, Wong D, Hill W, Hallmayer J (2004). Using self-report to identify the broad phenotype in parents of children with autistic spectrum disorders: A study using the Autism-Spectrum Quotient. Journal of Child Psychology and Psychiatry.

[CR10] Bölte S, Poustka F, Constantino JN (2008). Assessing autistic traits: Cross-cultural validation of the Social Responsiveness Scale (SRS). Autism Research.

[CR11] Caye A, Machado JD, Rohde LA (2013). Evaluating parental disagreement in ADHD diagnosis: Can we rely on a single report from home?. Journal of Attention Disorders.

[CR12] Central Office for Statistics (Centraal Bureau voor Statistiek). (2003). Voorburg, The Netherlands.

[CR13] Cicchetti DV (1994). Guidelines, criteria, and rules of thumb for evaluating normed and standardized assessment instruments in psychology. Psychological Assessment.

[CR14] Cohen J (1960). A coefficient of agreement for nominal scales. Educational and Psychological Measurement.

[CR15] Cohen J (1992). A power primer. Psychological Bulletin.

[CR16] Constantino JN, Gruber CP (2005). Social Responsiveness Scale.

[CR17] Constantino JN, Gruber CP (2012). Social Responsiveness Scale.

[CR18] Constantino JN, Lavesser PD, Zhang Y, Abbacchi AM, Gray T, Todd RD (2007). Rapid quantitative assessment of autistic social impairment by classroom teachers. Journal of the American Academy for Child and Adolescent Psychiatry.

[CR200] Corsello C, Hus V, Pickles A, Risi S, Cook EH, Leventhal BL (2007). Between a ROC and a hard place: Decision making and making decisions about using the SCQ.. Journal of Child Psychology and Psychiatry.

[CR19] De Los Reyes A (2013). Strategic objectives for improving understanding of informant discrepancies in developmental psychopathology research. Development and Psychopathology.

[CR20] De Los Reyes A, Kazdin AE (2005). Informant discrepancies in the assessment of childhood psychopathology: A critical review, theoretical framework, and recommendations for further study. Psychological Bulletin.

[CR21] De Vet HC, Terwee CB, Knol DL, Bouter LM (2006). When to use agreement versus reliability measures. Journal of Clinical Epidemiology.

[CR22] Dietz C, Swinkels SHN, Van Daalen E, Van Engeland H, Buitelaar JK (2006). Screening for autistic spectrum disorder in children aged 14–15 months. II: Population screening with the Early Screening of Autistic Traits Questionnaire (ESAT). Design and general findings. Journal of Autism and Developmental Disorders.

[CR23] Dirks MA, De Los Reyes A, Briggs-Gowan M, Cella D, Wakschlag LS (2012). Annual research review: Embracing not erasing contextual variability in children’s behavior—theory and utility in the selection and use of methods and informants in developmental psychopathology. Journal of Child Psychology and Psychiatry.

[CR24] Duhig AM, Renk K, Epstein MK, Phares V (2000). Interparental agreement on internalizing, externalizing, and total behaviour problems: A meta-analysis. Clinical Psychology: Science and Practice.

[CR25] Eaves LC, Wingert HD, Ho HH, Mickelson ECR (2006). Screening for autism spectrum disorders with the Social Communication Questionnaire. Developmental and Behavioral Pediatrics.

[CR26] Ehlers S, Gillberg C, Wing L (1999). A screening questionnaire for Asperger syndrome and other high-functioning autism spectrum disorders in school age children. Journal of Autism and Developmental Disorders.

[CR27] Fadnes LT, Taube A, Tylleskär T (2009). How to identify information bias due to self-reporting in epidemiological research. The Internet Journal of Epidemiology.

[CR28] Fernandopulle N (2011). Measurement of autism: A review of four screening measures. Indian Journal of Psychological Medicine.

[CR29] García-Primo P, Hellendoorn A, Charman T, Roeyers H, Dereu M, Roge B (2014). Screening for autism spectrum disorders: State of the art in Europe. European Child and Adolescent Psychiatry.

[CR30] Hoekstra RA, Bartels M, Cath DC, Boomsma DI (2008). Factor structure, reliability and criterion validity of the Autism-Spectrum Quotient (AQ): A study in Dutch population and patient groups. Journal of Autism and Developmental Disorders.

[CR31] Hurley RS, Losh M, Parlier M, Reznick JS, Piven J (2007). The Broad Autism Phenotype Questionnaire. Journal of Autism and Developmental Disorders.

[CR32] IBM Corp (2011). IBM SPSS Statistics for Windows, Version 20.0.

[CR33] Jepsen MI, Gray KM, Taffe JR (2012). Agreement in multi-informant assessment of behaviour and emotional problems and social functioning in adolescents with autistic and Asperger’s disorder. Research in Autism Spectrum Disorders.

[CR34] Kalyva E (2010). Multirater congruence on the Social Skills Assessment of children with Asperger syndrome: Self, mother, father, and teacher ratings. Journal of Autism and Developmental Disorders.

[CR35] Katz N, Petscher Y, Welles T (2009). Diagnosing attention-deficit hyperactivity disorder in college students: An investigation of the impact of informant ratings on diagnosis and subjective impairment. Journal of Attention Disorders.

[CR36] Kooij JJS, Boonstra AM, Swinkels SHN, Bekker EM, De Noord I, Buitelaar JK (2008). Reliability, validity, and utility of instruments for self-report and informant report concerning symptoms of ADHD in adult patients. Journal of Attention Disorders.

[CR37] Landis JR, Koch GG (1977). The measurement of observer agreement for categorical data. Biometrics.

[CR38] Langberg J, Epstein JN, Simon JO, Loren REA, Arnold LE, Hechtman L (2010). Parent agreement on ratings of children’s attention deficit/hyperactivity disorder and broadband externalizing behaviors. Journal of Emotional and Behavioral Disorders.

[CR39] Lord C, Rutter M, Le Couteur A (1994). Autism Diagnostic Interview-Revised: A revised version of a diagnostic interview for caregivers of individuals with possible pervasive developmental disorders. Journal of Autism and Developmental Disorders.

[CR40] Mascendaro PM, Herman KC, Webster-Stratton C (2012). Parent discrepancies in ratings of young children’s co-occuring internalizing symptoms. School Psychology Quarterly.

[CR41] Mattila MJ, Jussila K, Kuusikko S, Kielinen M, Linna SL, Ebeling H (2009). When does the Autism Spectrum Screening Questionnaire (ASSQ) predict autism spectrum disorders in primary school-aged children?. European Child and Adolescent Psychiatry.

[CR42] Maxwell CR, Parish-Morris J, Hsin O, Bush JC, Schultz RT (2013). The broad autism phenotype predicts child functioning in autism spectrum disorders. Journal of Neurodevelopmental Disorders.

[CR43] Moreno J, Silverman WK, Saavedra LM, Phares V (2008). Fathers’ ratings in the assessment of their child’s anxiety symptoms: A comparison to mothers’ ratings and their associations with parental symptomatology. Journal of Family Psychology.

[CR44] Multon KD, Salkind NJ (2010). Interrater reliability. Encyclopedia of research design.

[CR45] Norris M, Lecavalier L (2010). Screening accuracy of level 2 autism spectrum disorder rating scales: A review of selected instruments. Autism.

[CR46] Ozonoff S, Goodlin-Jones BL, Solomon M (2005). Evidence-based assessment of autism spectrum disorders in children and adolescents. Journal of Clinical Child and Adolescent Psychology.

[CR47] Podsakoff PM, MacKenzie SB, Lee JY, Podsakoff NP (2003). Common method biases in behavioural research: A critical review of the literature and recommended remedies. Journal of Applied Psychology.

[CR48] Posserud MB, Lundervold AJ, Gillberg C (2006). Autistic features in a total population sample of 7–9 year-old children assessed by the ASSQ (Autism Spectrum Screening Questionnaire). Journal of Child Psychology and Psychiatry.

[CR49] Renk K (2005). Cross-informant ratings of the behavior of children and adolescents: The “gold standard”. Journal of Child and Family Studies.

[CR50] Ruzich E, Allison C, Smith P, Watson P, Auyeung B, Ring H (2015). Measuring autistic traits in the general population: A systematic review of the Autism-Spectrum Quotient (AQ) in a non-clinical population sample of 6,900 typical adult males and females. Molecular Autism.

[CR51] Sasson NJ, Faso DJ, Parlier M, Daniels JL, Piven J (2014). When father doesn’t know best: Selective disagreement between self-report and informant report of the broad autism phenotype in parents of a child with autism. Autism Research.

[CR52] Sasson NJ, Lam KSL, Parlier M, Daniels JL, Piven J (2013). Autism and the broad autism phenotype: Familial patterns and intergenerational transmission. Journal of Neurodevelopmental Disorders.

[CR53] Seidman I, Yirmiya N, Milshtein S, Ebstein RP, Levi S (2012). The Broad Autism Phenotype Questionnaire: Mothers versus fathers of children with an autism spectrum disorder. Journal of Autism and Developmental Disorders.

[CR54] Smith SR (2007). Making sense of multiple informants in child and adolescent psychopathology: A guide for clinicians. Journal of Psychoeducational Assessment.

[CR55] Sollie H, Larsson B, Mørch WT (2013). Comparison of mother, father, and teacher reports of ADHD core symptoms in a sample of child psychiatric outpatients. Journal of Attention Disorders.

[CR56] Stratis EA, Lecavalier L (2015). Informant agreement for youth with autism spectrum disorder or intellectual disability: A meta-analysis. Journal of Autism and Developmental Disorders.

[CR57] Sucksmith E, Roth I, Hoekstra RA (2011). Autistic traits below the clinical threshold: Re-examining the broader autism phenotype in the 21st century. Neuropsychology Review.

[CR58] Swinkels SHN, Dietz C, Van Daalen E, Kerkhof IHGM, Van Engeland H, Buitelaar JK (2006). Screening for autistic spectrum in children aged 14–15 months. I: The development of the Early Screening of Autistic Traits Questionnaire (ESAT). Journal of Autism and Developmental Disorders.

[CR59] Van der Ende J, Verhulst FC, Tiemeier H (2012). Agreement of informants on emotional and behavioral problems from childhood to adulthood. Psychological Assessment.

[CR60] Van Wijngaarden-Cremers PJ, Van Eeten E, Groen WB, Van Deurzen PA, Oosterling IJ, Van der Gaag RJ (2014). Gender and age differences in the core triad of impairments in autism spectrum disorders: A systematic review and meta-analysis. Journal of Autism and Developmental Disorders.

[CR61] Wheelwright S, Auyeung B, Allison C, Baron-Cohen S (2010). Defining the broader, medium and narrow autism phenotype among parents using the Autism Spectrum Quotient (AQ). Molecular Autism.

[CR62] Zwaigenbaum L, Bryson S, Lord C, Rogers S, Carter A, Carver L (2009). Clinical assessment and management of toddlers with suspected autism spectrum disorder: Insights from studies of high-risk infants. Pediatrics.

